# The differing effects of a dual acting regulator on SIRT1

**DOI:** 10.3389/fmolb.2023.1260489

**Published:** 2023-08-30

**Authors:** Yujin Hur, Johnson Huynh, Emily Leong, Reena Dosanjh, Annemarie F. Charvat, My H. Vu, Zain Alam, Yue Tong Lee, Christiane C. Cabreros, Emma C. Carroll, Greg L. Hura, Ningkun Wang

**Affiliations:** ^1^ Department of Chemistry, San José State University, San José, CA, United States; ^2^ Department of Pharmaceutical Chemistry, University of California, San Francisco, CA, United States; ^3^ Molecular Biophysics and Integrated Bioimaging Division, Lawrence Berkeley National Laboratory, Berkeley, CA, United States

**Keywords:** allosteric regulation, enzyme kinetics, deacetylase, conformational change, SIRT1

## Abstract

SIRT1 is an NAD^+^-dependent protein deacetylase that has been shown to play a significant role in many biological pathways, such as insulin secretion, tumor formation, lipid metabolism, and neurodegeneration. There is great interest in understanding the regulation of SIRT1 to better understand SIRT1-related diseases and to better design therapeutic approaches that target SIRT1. There are many known protein and small molecule activators and inhibitors of SIRT1. One well-studied SIRT1 regulator, resveratrol, has historically been regarded as a SIRT1 activator, however, recent studies have shown that it can also act as an inhibitor depending on the identity of the peptide substrate. The inhibitory nature of resveratrol has yet to be studied in detail. Understanding the mechanism behind this dual behavior is crucial for assessing the potential side effects of STAC-based therapeutics. Here, we investigate the detailed mechanism of substrate-dependent SIRT1 regulation by resveratrol. We demonstrate that resveratrol alters the substrate recognition of SIRT1 by affecting the *K*_M_ values without significantly impacting the catalytic rate (*k*_cat_). Furthermore, resveratrol destabilizes SIRT1 and extends its conformation, but the conformational changes differ between the activation and inhibition scenarios. We propose that resveratrol renders SIRT1 more flexible in the activation scenario, leading to increased activity, while in the inhibition scenario, it unravels the SIRT1 structure, compromising substrate recognition. Our findings highlight the importance of substrate identity in resveratrol-mediated SIRT1 regulation and provide insights into the allosteric control of SIRT1. This knowledge can guide the development of targeted therapeutics for diseases associated with dysregulated SIRT1 activity.

## 1 Introduction

SIRT1 is a mammalian NAD^+^-dependent Class III lysine deacylase. A large variety of proteins governing different cellular functions have been identified as substrates for SIRT1, implicating its involvement in many important cellular pathways, including glucose metabolism, cellular apoptosis, and neurodegeneration (Qiu et al., 2010; Wang et al., 2010; Yi and Luo, 2010). Allosterically regulating the activity of SIRT1 has been explored as a therapeutic approach for treating several diseases such as Alzheimer’s disease and Type II diabetes (Sinclair and Guarente, 2014; Manjula et al., 2021). However, information is still lacking regarding the detailed mechanism for SIRT1 regulation, hampering further progress in the development of therapeutics and understanding SIRT1 regulation in the cell.

Various small molecules have been identified as allosteric SIRT1 activators, termed Sirtuin Activating Compounds (STACs). While there are various high-quality studies on the effects of STACs on SIRT1 activity, the yielded results can be limited, as oftentimes only one or two canonical peptide substrates (such as Ac-p53W) are used in the activity assays. A survey on the effect of resveratrol, a well-studied STAC (Ungurianu et al., 2023), on SIRT1 activity towards 6,802 different acetylated peptide substrates has found that resveratrol can act either as an activator, have no effect, or act as an inhibitor against SIRT1, dependent on the peptide substrate sequence (Lakshminarasimhan et al., 2013). This study showed that resveratrol acts as a double agent rather than a simple activator, and implies serious consequences. As some STACs are progressing in preclinical and clinical trials (Curry et al., 2021), understanding the nature of this dual behavior will be crucial in assessing their potential side and off-target effects in the body. In the survey study on resveratrol’s substrate-dependent effect on SIRT1, of particular interest to us is the >300 peptide substrates where resveratrol inhibits SIRT1 activity. While the current understanding of the field is that resveratrol activates SIRT1 by enhancing substrate binding for “loose substrates” (Hou et al., 2016), the mechanism by which resveratrol acts as an inhibitor for SIRT1 has not been explored.

**FIGURE 1:**
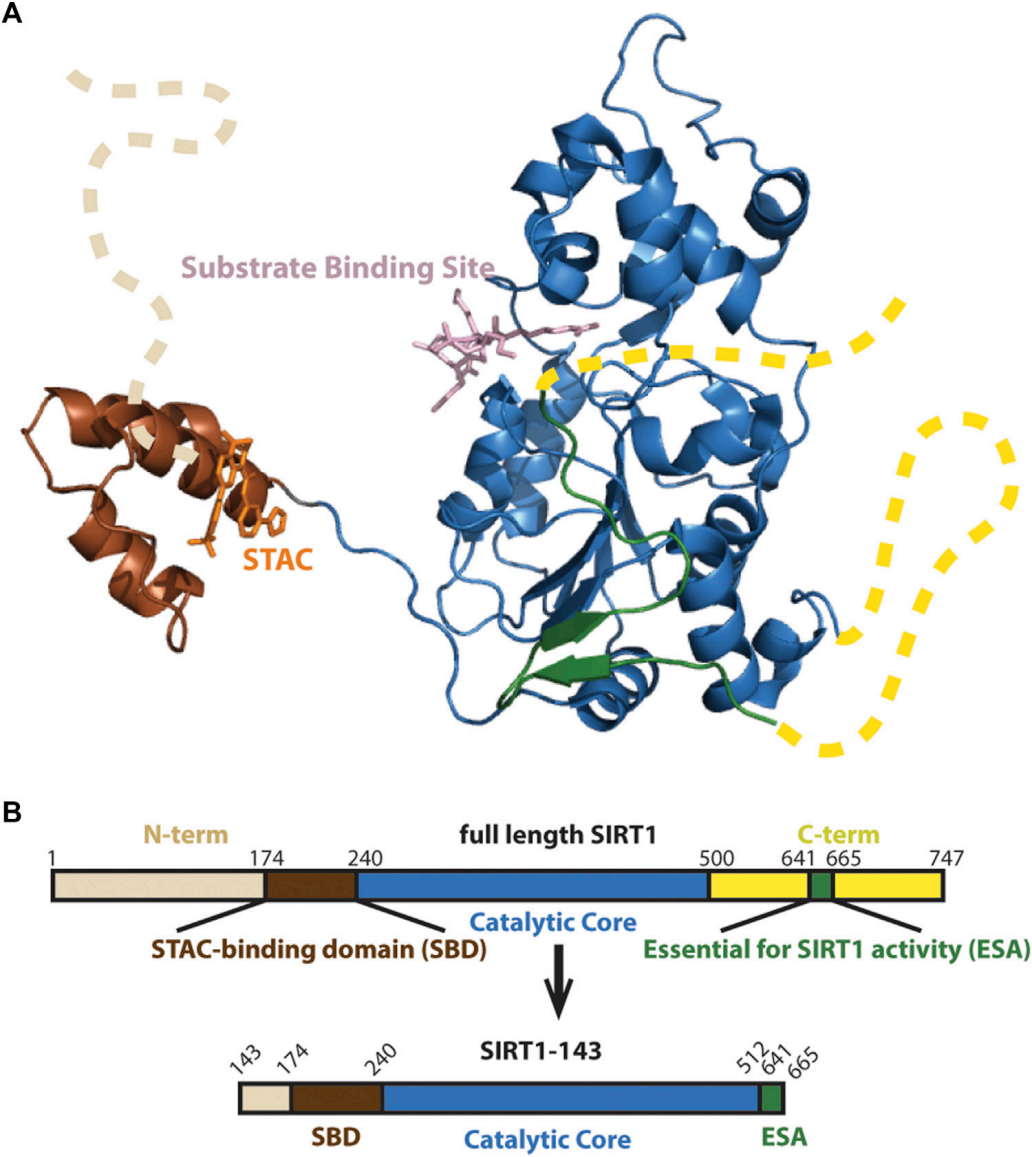
**(A)** Structure of SIRT1 (PDB 4ZZJ) with the catalytic core shown in blue, the N-terminal’s SBD is shown in brown and C-terminal’s Essential for SIRT1 Activity (ESA) domain shown in green completing the beta sheets at the Rossman fold. Unstructured N- and C-terminals shown as dashed lines. **(B)** Schematic of SIRT1-143 design, consisting of residues 143–512 and the region 641–665.

The architecture of the human SIRT1 includes a structured catalytic core (Davenport et al., 2014) that is highly conserved in the Sirtuin family (Sanders et al., 2010), and flanking N- and C-terminal domains that are partially unstructured and potentiate deacetylase activity (Figure 1A) (Pan et al., 2012; Meledin et al., 2013). While the structure and function of the catalytic core is relatively well understood (Autiero et al., 2009; Zhao et al., 2013; Davenport et al., 2014), the functions of the protein’s flanking N-terminal and C-terminal unstructured regions are less clear. In recent years, studies have shown that the N-terminal region, especially a three-helix bundle termed the STAC binding domain (SBD), is important for enzyme activity and STAC-dependent activation (Kang et al., 2011; Dai et al., 2015; Ghisays et al., 2015; Krzysiak et al., 2018).

We hypothesized that the conformational change of the SBD relative to the catalytic core plays a central role in conveying activation or inhibition of SIRT1 by small-molecule regulators. Here, using resveratrol as a chemical biology tool, we dissect the mechanism of this substrate-dependent regulation through comparing the kinetics, stability, conformation and binding properties of SIRT1 in complex with peptide substrates p53W(K382), p53(K382), histone H4 (K9), histone H3 (K14) and casein kinase 1 alpha 1 (CSNK) (K8). We found that resveratrol both activates and inhibits SIRT1 through destabilizing the protein and extending the protein conformation. However, resveratrol likely affects SIRT1 conformation in a different manner when acting as an activator versus an inhibitor.

## 2 Materials and methods

### 2.1 Protein expression and purification

In order to express his-tagged and SUMO (small ubiquitin-like modifier) solubility tagged hSIRT1-143 constructs, chemically competent *E. coli* BL21 (DE3) cells containing the pET28-smt3-hSIRT1-143 plasmid were transferred into a 5 mL culture tube with LB and Kanamycin, and shaken overnight at 37°C, 250 rpm. The next day, an overnight culture was diluted 1:100 (v/v) into 1 L of Terrific Broth that included 35 μg/mL of Kanamycin. The culture was incubated at 37°C, 220 rpm, until Abs_600_ was around 0.70 before being induced with isopropyl-1-thio-D-galactopyranoside (IPTG) at a final concentration of 1 mM. The culture was then grown at 16°C for 16–18 h. The cells were harvested by 15 min of centrifugation at 4,500 g, and stored as a dry cell pellet at −80°C.

To purify the protein, the cell pellet was thawed, and homogenized in lysis buffer (50 mM Tris pH 8.0, 150 mM NaCl, 20 mM imidazole, 10% glycerol, 100 M PMSF, 3 mM β-mercaptoethanol), and lysed by sonication. The Ni-NTA resin was incubated with the cleared lysate and washed with lysis buffer. The His-SUMO-hSIRT1 protein was eluted using elution buffer (50 mM Tris pH 8.0, 150 mM NaCl, 200 mM imidazole, 10% glycerol, and 3 mM -mercaptoethanol). Elution fractions containing target protein were combined, then Ulp1 (a SUMO protease) was added to the mixture to cleave off the SUMO solubility tag. The solution then underwent overnight dialysis into lysis buffer at 4°C. The cleaved hSIRT1 was further purified by Size Exclusion Chromatography (SEC) (Hiprep 16/20 Sephacryl S-160) using the ÄKTA™ Start FPLC system and eluted with storage buffer (50 mM Tris pH 8.0, 150 mM NaCl, 10% glycerol, 1 mM DTT). Fractions containing monomeric, cleaved hSIRT1-143 as determined by SDS-PAGE were combined, aliquoted, and flash frozen using liquid nitrogen. These fractions were then stored at −80°C for later use. Bradford assays and BCA assays were used to determine protein concentration.

### 2.2 Enzyme-coupled assays

Assays were conducted in accordance with a prior publication (Smith et al., 2009). Peptide substrates were 11 or 13-mer peptides with acetylated lysine at the middle residue position, synthesized and purified by Elim BioPharm (Hayward, CA) to a purity of 90%. The following reagents were used in the assay mixtures: 5–640 μM peptide substrate, 640 μM NAD^+^, 2 mM MPB-PncA (nicotinamidase), 20 mM phosphate buffer pH 7.6, 3 mM α-ketoglutarate, 1 mM DTT, 0.2 mM NADPH, and 6.7 units of glutamate dehydrogenase. Then, 0.2–1 μM of the SIRT1 enzyme was added to start the reactions. SIRT1 reactions were conducted in a transparent, flat-bottomed, 96-well plate with a final volume of 150 μL per well and read with a BioTek™ Synergy HTX Multimode Reader. A static read was first taken to ensure that the absorbance at 340 nm did not change. After SIRT1 was added, absorbance at 340 nm was measured to quantify the rate of NADPH depletion for a duration of 10 min. Initial rates of the reaction were calculated using 6.22 mM^-1^cm^-1^ as the extinction coefficient for NADPH and fitting the slope of the linear component of the reaction. The initial velocity of the reaction absent the peptide substrate, which served as a background control, was subtracted from the initial velocities of all other reactions. By fitting the initial rate data to the Michaelis-Menten equation in GraphPad Prism, the values for *k*_cat_ and *K*_M_ were obtained.

### 2.3 Differential scanning fluorimetry

Differential Scanning Fluorimetry experiments were performed in a 384-well plate using a qTOWER real-time PCR thermal cycler (Analytik Jena). Fluorescence intensity was measured in the TAMRA channel (Ex: 535 nm Em: 580 nm) over a period of 1 h with a 1°C increase temperature ramp per cycle in “Normal” mode. 25 μM hSIRT1 bound to an excess of ADPr (640 µM), 200 µM resveratrol, and 500—5,000 µM peptide substrate (as appropriate for each condition based on kinetics data) was added to each well. 1:100 SYPRO^®^ Orange (Millipore) dye diluted in buffer was also added to each well. All measurements were performed in triplicate, with buffer-only controls containing ADPr, resveratrol, and peptide performed for all conditions. T_ma_ was calculated using the first derivative method and fitting a Lorentzian distribution to the first derivative curve (T_ma_ = center of curve/inflection point) in GraphPad Prism (Wu et al., 2020).

### 2.4 Small angle X-ray scattering (SAXS) experiments

SAXS samples were prepared with 40 μM of hSIRT1-143 in storage buffer (50 mM Tris pH 8.0, 150 mM NaCl, 1 mM DTT). All samples contained 640 μM of NAD^+^ analog ADPr (adenosine diphosphate ribose) except for the apo SIRT1 sample. Varying concentrations of different peptide substrates (500–5,000 μM) were added to the different samples. The concentration of peptide substrate added was determined based on previous kinetics assays to ensure that SIRT1 is mostly saturated with the peptide. 200 μM resveratrol was added to the “+ Resveratrol” samples to best approximate the conditions in the enzyme activity assays in this study. A “blank” solution for each sample including all components in the samples (substrates, resveratrol, etc.) except for SIRT1 was used for background subtraction.

At the Lawrence Berkeley National Laboratory (CA, United States), SAXS data were obtained using the Advanced Light Source SIBYLS beamline 12.3.1 in the high-throughput mode (HT-SAXS) (Classen et al., 2013). With an X-ray wavelength of 1.216 Å and a sample-to-detector distance of 2070 mm, the scattering vector, q, varied from 0.01 to 0.45. Where 2θ is the scattering angle, the scattering vector is defined as q = 4π sin θ/λ. The experiments were conducted at a temperature of 20°C. The sample was placed for 10 s with the detector framing at 0.3 s to optimize the signal using the SAXS FrameSlice program (https://bl1231.als.lbl.gov/ran). ATSAS 3.0 Primus software was utilized to plot and analyze the combined SAXS (Manalastas-Cantos et al., 2021).

### 2.5 Binding affinity measurements

A quartz cuvette from Starna Cells (Atascadero, CA) containing 150 μL of 2 μM of hSIRT1-143 protein (20 mM phosphate buffer, pH 7.6) was prepared and resveratrol was titrated in at a range of concentrations between 0.7–422.6 μM. Using a Cary Eclipse Fluorescence Spectrometer with excitation at 285 nm and emission between 300 and 500 nm, the intensity of intrinsic tryptophan fluorescence was measured. Fluorescence intensity was recorded at the peak of emission spectra (330–337 nm for most complexes and 359 nm for the complex with Ac-p53W). To account for dilution effects and background fluorescence from the hSIRT1 protein (*F*_*0*_), the fluorescence intensity (*F*_*i*_) was adjusted in a way that *ΔF = F*_*0*_ *—F*_*i*_. GraphPad Prism software was used to fit the data using a 1:1 binding model of hSIRT1 to resveratrol to determine the apparent equilibrium dissociation constant (*K*_D_). The observed fluorescence fraction increase (*ΔF/F*_*0*_) was plotted as a function of resveratrol concentration, and a binding isotherm that reflects ligand depletion (Eq. 1) was used. hSIRT1-143 protein and resveratrol total concentrations are represented by the parameters a and x, respectively. Observed relative fluorescence quenching at any resveratrol concentration is represented by y. Maximum and minimum observed relative fluorescence quenching values are represented by b and c, respectively.

$$y=c+\left(b-c\right)\times \frac{\left(\left(K_{D}+a+x\right)-\sqrt{\left.{\left(K_{D}+a+x\right)}^{2}-4\times a\times x\right)}\right.}{2\times a}$$ (1)

## 3 Results

### 3.1 Resveratrol alters *K*_M_ in both activation and inhibition scenarios

We employed a hSIRT1-143 construct for the biochemical investigations that was based on earlier research (Cao et al., 2015), which included the SBD and catalytic core as well as the C-terminal ESA (Essential for Sirtuin Activity) domain (Figure 1B). This construct has been used in investigations on resveratrol-based allosteric interactions involving the N-terminal region and has been proven to have similar activity as full-length hSIRT1 (Dai et al., 2015).

**FIGURE 2:**
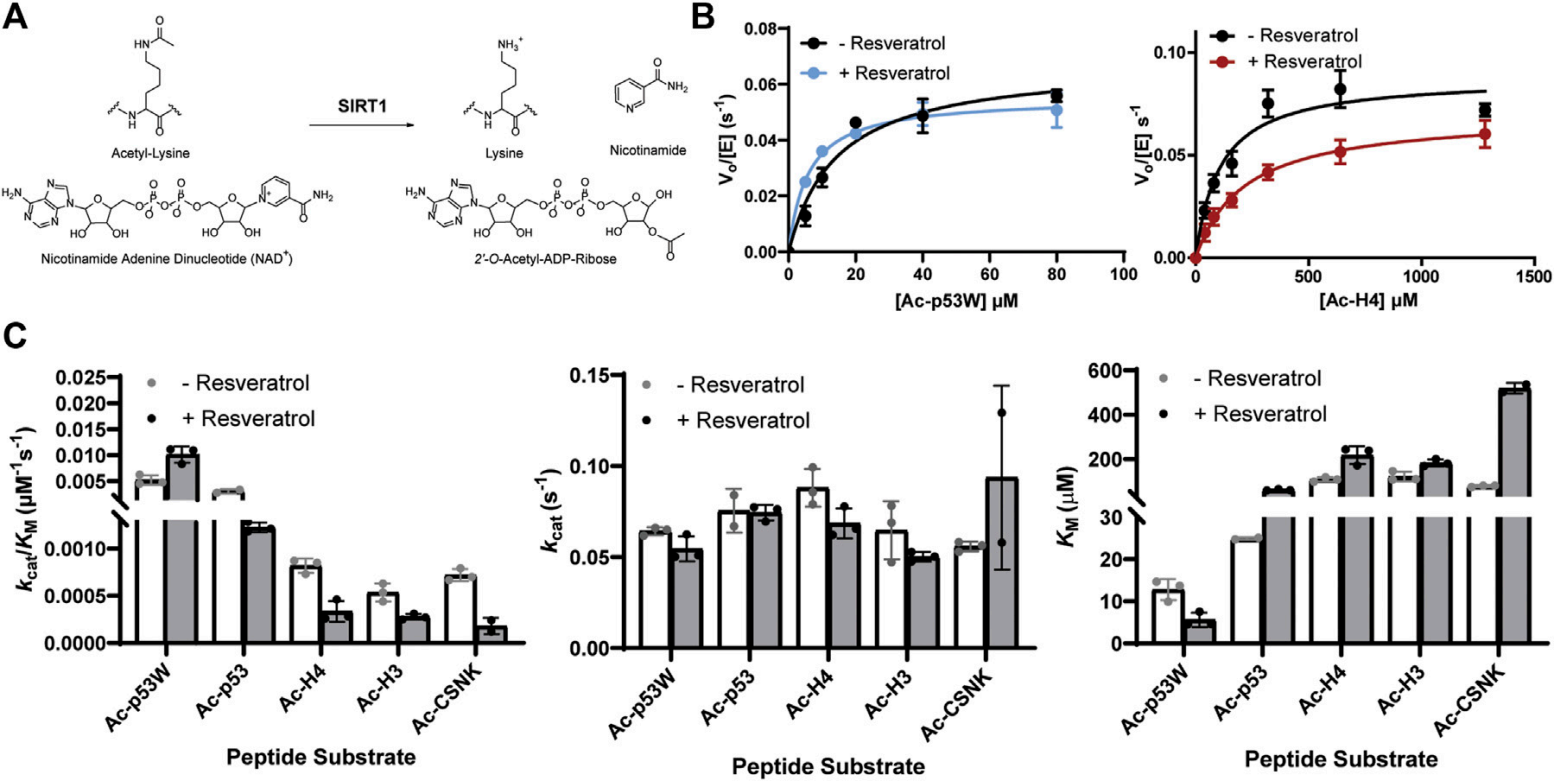
**(A)** Reaction scheme of SIRT1. **(B)** Representative enzyme kinetics curves for SIRT1 activity against Ac-p53W and Ac-H4 with and without the addition of resveratrol. Resveratrol acts as an activator for SIRT1 activity towards Ac-p53W and acts as an inhibitor for SIRT1 activity towards Ac-H4. **(C)** Michaelis-Menten parameters for SIRT1 activity against different peptide substrates with and without resveratrol. All kinetics data were collected in at least triplicates and fit with GraphPad Prism. The average and SEM are reported. All *K*_M_ and *k*_cat_/*K*_M_ values have a *p*-value <0.05 between—resveratrol and + resveratrol samples. None of the *k*_cat_ values have a *p*-value <0.05 between—resveratrol and + resveratrol samples.

Previous work that identified resveratrol as both inhibitor and activator mainly used end-point assays. To gain more insight into the mechanism of this allosteric regulation, we conducted enzyme kinetics assays (Smith et al., 2009) to obtain the Michaelis-Menten parameters for SIRT1’s activity against the different peptide substrates with and without resveratrol (Figures 2A–C; Table 1). Amongst the five peptide substrates that were chosen based on previous studies, we determined that resveratrol increased overall SIRT1 activity (*k*_cat_/*K*_M_) towards Ac-p53W by 2-fold, and decreased overall SIRT1 activity towards Ac-p53, Ac-H4, Ac-H3 and Ac-CSNK by 30%–50%. Resveratrol did not have significant effects on the *k*_cat_ values towards any of the various peptide substrates. In contrast, there were statistically significant changes in the *K*_M_ values toward all the peptide substrates. We observed a 2-fold decrease in *K*_M_ towards Ac-p53W where resveratrol acts as an activator and 2-6 fold increases in *K*_M_ towards Ac-p53, Ac-H4, Ac-H3 and Ac-CSNK, where resveratrol acts as an inhibitor (Figure 2C; Table 1). This indicates that substrate recognition was the driving force that caused the resveratrol-dependent change in overall enzyme efficiency.

**TABLE 1:** Michaelis-Menten kinetics parameters of hSIRT1-143 activity against various peptide substrates with and without the addition of 200 μM resveratrol. All enzyme kinetics data were obtained in triplicates or duplicates. The Michaelis-Menten parameters were fit with GraphPad Prism and the average and SEM are reported. All *K*_M_ and *k*_cat_/*K*_M_ values have a *p*-value < 0.05 between—resveratrol and + resveratrol samples. None of the *k*_cat_ values have a *p-*value < 0.05 between—resveratrol and + resveratrol samples.

Peptide substrates	Resveratrol	*k*_cat_ (s^-1^)	*K*_M_ (μM)	*k*_cat_/*K*_M_ (μM^-1^s^-1^)
Ac-p53W (^376^STSRHKK^Ac^WMFKTE^389^)	-	0.06 ± 0.001	13 ± 1.4	0.005 ± 0.0005
	+	0.06 ± 0.004	6 ± 1.0	0.01 ± 0.0009
Ac-p53 (^376^STSRHKK^Ac^LMFKTE^388^)	-	0.08 ± 0.009	25 ± 0.2	0.003 ± 0.00003
	+	0.07 ± 0.002	61 ± 1.6	0.002 ± 0.0003
Ac-H4 (^3^VRGKAGK^Ac^GLGKGG^15^)	-	0.09 ± 0.006	108 ± 5.5	0.001 ± 0.0004
	+	0.07 ± 0.005	217 ± 23.2	0.0005 ± 0.0001
Ac-H3 (^9^KSTGGK^Ac^APRKQ^19^)	-	0.06 ± 0.009	121 ± 12.6	0.0005 ± 0.0003
	+	0.05 ± 0.002	183 ± 8.9	0.0003 ± 0.00006
Ac-CSNK (^2^ASSSGSK^Ac^AEFIVG^14^)	-	0.06 ± 0.002	78 ± 2.2	0.0007 ± 0.0002
	+	0.09 ± 0.036	519 ± 16.9	0.0005 ± 0.0001

### 3.2 Resveratrol decreases the thermal stability of SIRT1 to a larger extent in inhibition scenarios

**FIGURE 3:**
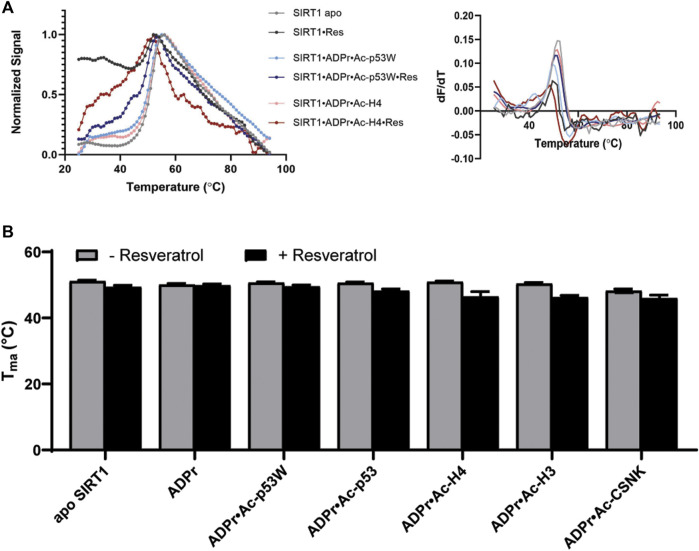
**(A)** Representative DSF melting curves and their first derivatives for apo SIRT1 and SIRT1•substrate complexes with and without the addition of resveratrol. Resveratrol acts as an activator for SIRT1 activity towards Ac-p53W and acts as an inhibitor for SIRT1 activity towards Ac-H4. **(B)** Bar graph of T_M_ values for various SIRT1 complexes with and without the addition of resveratrol. Data are collected in triplicate or duplicate, the SIRT1•ADPr•Ac-H3 + Resveratrol data set is only a single data set. The center of the curve/inflection point and SEM of fit are reported.

We used differential scanning fluorimetry (DSF) with SYPRO^®^ Orange as the dye to evaluate the effect of resveratrol on the thermal stability of the different SIRT1•substrate complexes (Wu et al., 2023). In addition to the peptide substrate, we also added ADPr (Adenosine diphosphate ribose), which is an analog of NAD^+^, a co-substrate of SIRT1. The melting temperatures were obtained by the first derivative method for calculating T_ma_ (Figure 3A). This enabled the comparison of the relative T_ma_ values for various SIRT1•substrate complexes with and without the addition of resveratrol.

The T_ma_ value of the apo hSIRT1-143 was 51.1°C ± 0.3°C, which is in the same range as previous studies (Kalous et al., 2016). In general, resveratrol destabilized apo SIRT1, SIRT1 in complex with just ADPr, and all of the SIRT1•substrate complexes regardless of it acting as an activator or inhibitor (ΔT_ma_ = 1°C–4°C, respectively) (Figure 3B; Table 2). However, resveratrol had a larger effect on the stability of SIRT1 complexes under inhibition scenarios (Ac-p53, Ac-H4, Ac-H3 and Ac-CSNK), decreasing the melting temperature by 2°C–4°C, whereas it only decreased the melting temperature by 1°C for the SIRT1•ADPR•Ac-p53W complex where it acts as an activator. In the absence of substrate, resveratrol decreased the T_ma_ of apo SIRT1 by 1.7°C, and decreased the T_ma_ of SIRT1 in complex with ADPr by only 0.2°C. It is possible that resveratrol inherently affects SIRT1 stability, the addition of ADPr “protects” resveratrol from destabilizing the protein, but the addition of the peptide substrates renders SIRT1 more flexible or susceptible to resveratrol destabilization.

**TABLE 2:** The melting temperatures of SIRT1 in complex with different peptide substrates and ADPr, an NAD^+^ analog, with and without the addition of 200 μM resveratrol as obtained by fitting the first derivative of the DSF data with a Lorentzian function. Data are collected in triplicate or duplicate, the SIRT1•ADPr•Ac-H3 + Resveratrol data set is only a single data set. The center of the curve/inflection point and SEM of fit are reported.

Protein complex	T_ma_ (°C) − res	T_ma_ (°C) + res
apo SIRT1	51.1 ± 0.27	49.4 ± 0.48
SIRT1•ADPr	50.1 ± 0.34	49.9 ± 0.44
SIRT1•ADPr•Ac-p53W	50.7 ± 0.27	49.5 ± 0.46
SIRT1•ADPr•Ac-p53	50.6 ± 0.009	48.2 ± 0.54
SIRT1•ADPr•Ac-H4	50.9 ± 0.26	46.5 ± 1.49
SIRT1•ADPr•Ac-H3	50.4 ± 0.32	46.3 ± 0.55
SIRT1•ADPr•Ac-CSNK	48.2 ± 0.57	46.0 ± 0.94

### 3.3 Resveratrol affects SIRT1 conformation in differing aspects in the two scenarios

To take a closer look at the effect of resveratrol on SIRT1 conformation in the context of different peptide substrates, we conducted small angle X-ray scattering (SAXS) experiments. Briefly, SIRT1 was combined with ADPr as well as the different peptide substrates at various concentrations, these concentrations ensured the peptides were saturating SIRT1 based on our kinetics results. SAXS profiles were collected for these complexes with and without the addition of 200 µM resveratrol. A “blank” solution including all components in the samples (substrates, resveratrol, etc.) except for SIRT1 was used for background subtraction to ensure the SAXS data only reflected the conformation of SIRT1 and not of the free peptide substrates.

**FIGURE 4:**
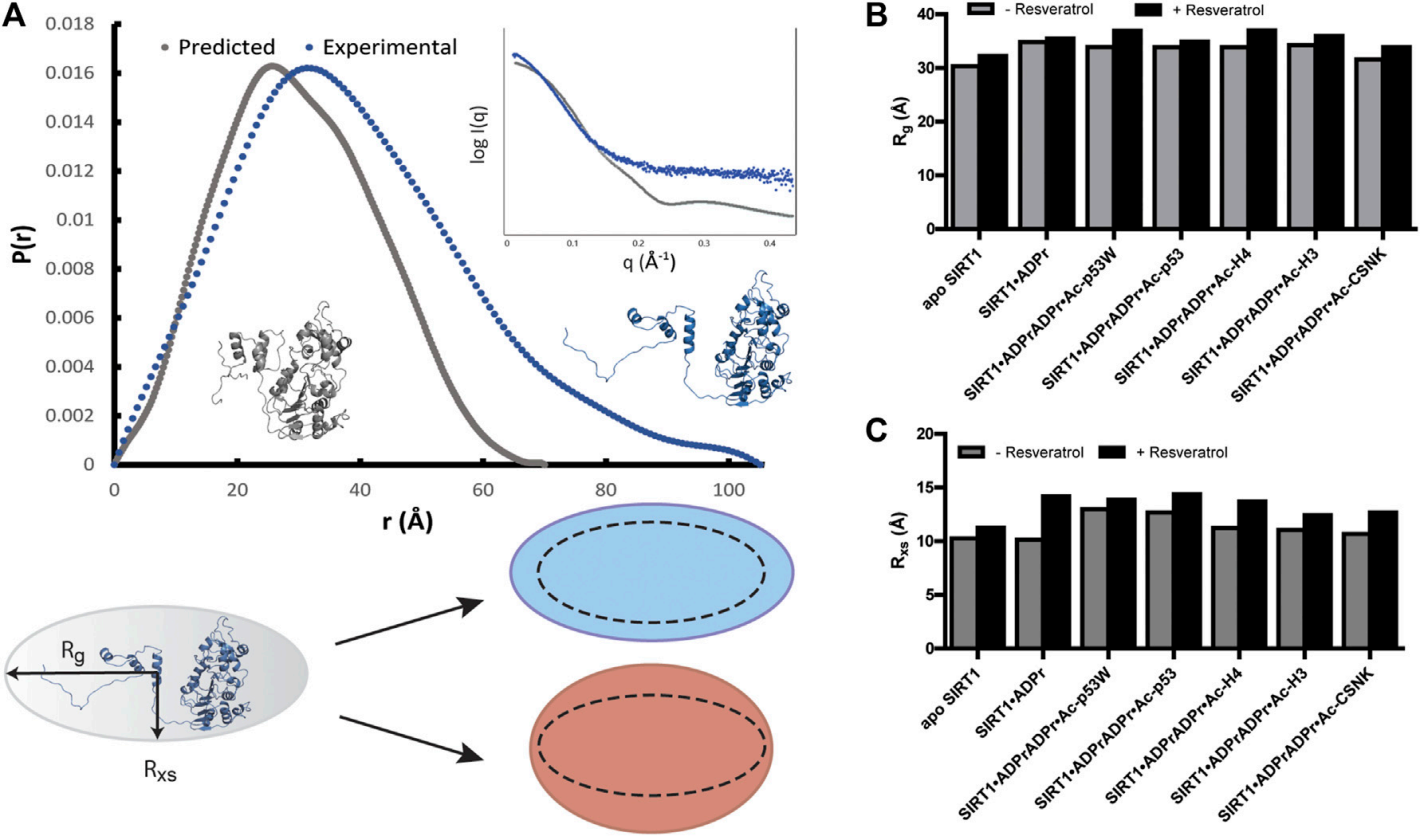
**(A)** p(r) functions computed from the crystal structure of hSIRT1-143 (PDB 5BTR) in gray and analyzed from experimental SAXS profiles of hSIRT1-143 in solution shown in blue. The corresponding structures suggest a possible scenario for the extended conformation. Inset shows the overlay of theoretical and experimental SAXS profiles. Cartoon shows the difference between R_g_ and R_xs_ and the possible overall conformation of SIRT1 with the 1:3 ratio of R_xs_:R_g_ along with schematics of how the change in both parameters might be manifested under activation (blue) and inhibition (red) scenarios. **(B)** Bar graphs comparing the R_g_ values for SIRT1 when in different complexes with and without resveratrol. **(C)** Bar graphs comparing the R_xs_ values for SIRT1 when in different complexes with and without resveratrol.

In all the samples, SIRT1 presented as monomeric based on the molecular weight analysis (Manalastas-Cantos et al., 2021). However, the experimental SAXS profile of apo SIRT1 as well as the other SIRT1 complexes were significantly different from the computed SAXS profile of SIRT1-143 using the PDB structure 5BTR and the FOXS server (Figure 4A) (Schneidman-Duhovny et al., 2016). The predicted radius of gyration (R_g_) for SIRT1-143 is 23 Å, however the experimental R_g_ values were all >30 Å, suggesting that in solution, SIRT1 is much more extended than the well-folded crystal structure conformation. Additionally, the SAXS data suggest that SIRT1 assumes a more rod-like conformation, where the radius of gyration ranged from 31 to 37Å and the radius of cross-section (R_xs_) ranged from 10 to 14 Å (Figure 4 B, C; Supplementary Table S1). The *p*(r) function for the samples, which represents the histogram of distances between pairs of points within the particle (Putnam et al., 2007), shows that besides a compact conformation with a diameter at around 30–35 Å, a small population of SIRT1 also exists as an extended conformation with a diameter of >100 Å (Figure 4A). This is consistent with the fact that previous structures of SIRT1 show that the loop connecting the SBD and the catalytic core is flexible, and the conformation of the SBD relative to the catalytic core fluctuates greatly (Cao et al., 2015; Dai et al., 2015), as well as previous studies that showed the three helices of the SBD could become disordered and less bundled when bound to inhibitor proteins (Krzysiak et al., 2018).

Upon comparison of the SAXS profiles with and without resveratrol, it was clear that the addition of resveratrol extended the conformation of SIRT1 regardless of the peptide substrate sequence. However, R_xs_ seemed to increase in higher percentage (12%–22%) than R_g_ (3%–9%) in the conditions where resveratrol acts as an inhibitor (substrates Ac-p53, Ac-H4, Ac-H3, Ac-CSNK), whereas R_xs_ increased in similar percentage (7%) compared R_g_ (9%) when SIRT1 was complexed with Ac-p53W, the condition where resveratrol acts as an activator (Figures 4B, C; Supplementary Table S1). Based on our *p*(r) functions, it seems that the addition of resveratrol affects the extended population at approximately 100 Å distance (Supplementary Figure S4), but as this is a small population in our sample, more detailed interpretation is hindered by the low signal to noise ratio for this region.

### 3.4 Resveratrol binds to SIRT1 with lower affinity in activation scenarios

We hoped to shed more light on the reason behind how resveratrol can act as both an activator and inhibitor based on the peptide substrates. Previous work has shown that the catalytic core of SIRT1 undergoes conformational change when bound to its substrates (Davenport et al., 2014). We hypothesized that different substrates would induce slightly different conformational changes, thus interacting differently with resveratrol. To test this, we quantified the binding affinity of resveratrol to the various SIRT1•substrate complexes using a tryptophan quenching assay (Yammine et al., 2019).

**FIGURE 5:**
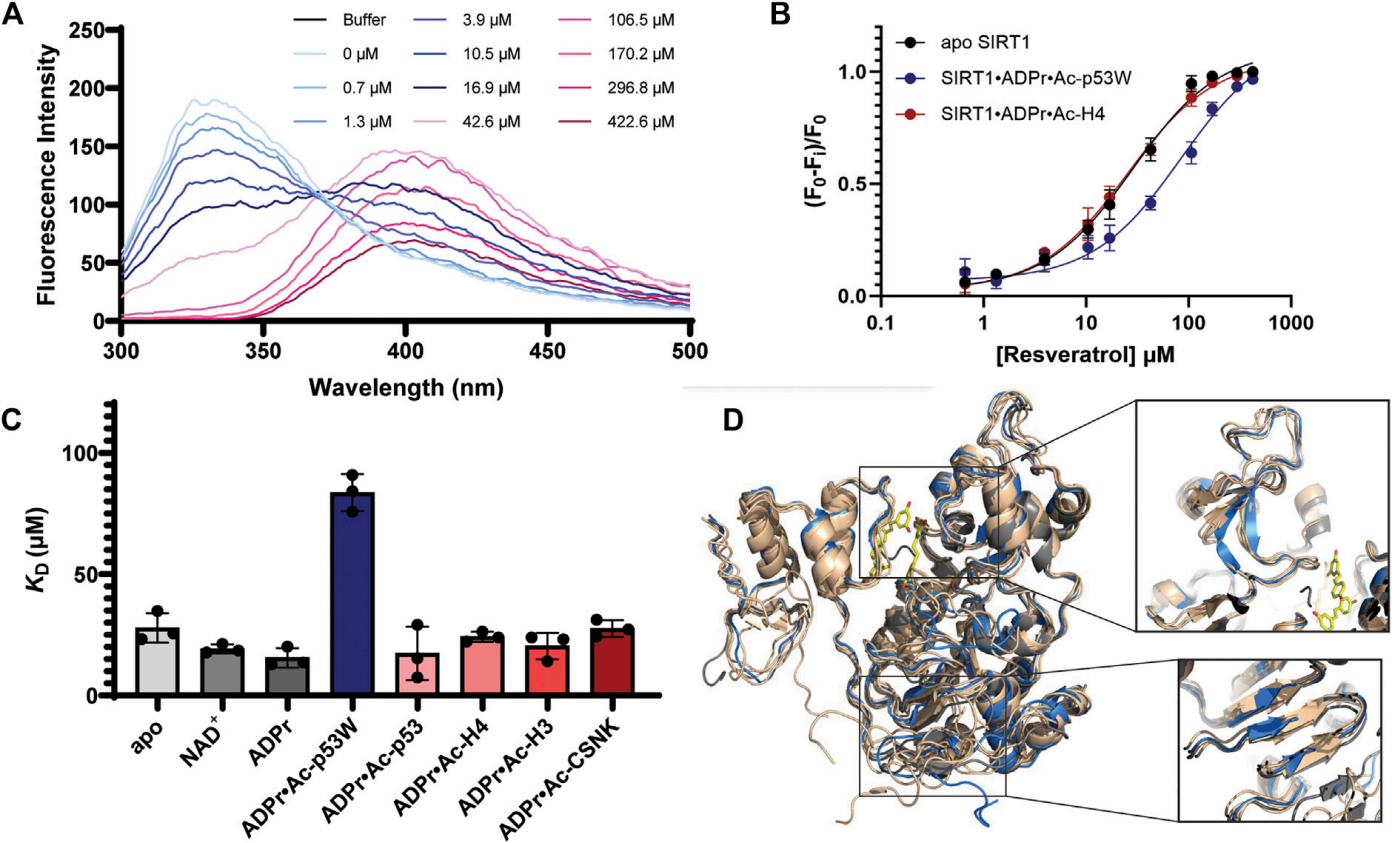
**(A)** Representative fluorescence spectra of SIRT1 upon titration of resveratrol from 0.7 μM to 422.6 μM showing the decrease of the peak at 330 nm. **(B)** Representative binding isotherms from GraphPad Prism for resveratrol binding to apo SIRT1 and SIRT1 in complex with substrates. Resveratrol acts as an activator for SIRT1 activity towards Ac-p53W and acts as an inhibitor for SIRT1 activity towards Ac-H4. **(C)** Bar graph of binding affinities between resveratrol and SIRT1 complexed with different substrates. All measurements were performed in triplicate and the average and SEM are reported. Only the binding affinity of resveratrol to the SIRT1•ADPr•Ac-p53W complex is statistically significant different (*p-*value < 0.05) from the binding affinity of resveratrol to apo SIRT1. **(D)** Overlay of CABS-dock structures of hSIRT1-143 where the different peptide substrates were submitted as peptide ligands for docking. The original 5BTR structure is shown in gray, all the structures resulting from binding to a peptide substrate where resveratrol acts as an inhibitor are shown in tan, the structure resulting from binding to Ac-p53W, where resveratrol acts as an activator, is shown in blue. The small molecules in yellow are the 3 resveratrol molecules in 5BTR. Two regions where the blue structure shows significant deviation from all other structures are zoomed in.

The hSIRT1-143 construct contains two tryptophan residues, W176 and W221, both of which are located in the SBD region, where resveratrol binds. The intrinsic fluorescence of tryptophan was markedly quenched after resveratrol was titrated in (Figure 5A). This phenomenon allowed us to ascertain the binding affinity between the various SIRT1•substrate complexes and resveratrol (Figure 5B). The resulting *K*_D_ values are shown in Figure 5C and Table 3. Similar to the DSF and SAXS experiments, we used ADPr as a non-reacting substitute for NAD^+^. The *K*_D_ values between resveratrol and SIRT1•NAD^+^ and SIRT1•ADPr were comparable, suggesting ADPr likely had the same effect on SIRT1 conformation as NAD^+^ and was a serviceable substitution. Our *K*_D_ values were comparable to those obtained by isothermal calorimetry (ITC) in previous studies (Zhang et al., 2021). Our *K*_D_ for resveratrol and apo SIRT1 was 28 μM, slightly lower than a literature *K*_D_ of 50 μM. This is possibly due to the different SIRT1 constructs used, our construct had additional residues 143–182 compared to the construct in literature that started at residue 183. As resveratrol is known to bind to the SBD region (174–240) (Cao et al., 2015), it is not unreasonable to expect an additional 40 amino acids adjacent to this region could strengthen the interaction. Our *K*_D_ for resveratrol and the SIRT1•ADPr•Ac-p53 complex was 16 μM, which agrees well with the literature value of 14 μM (Zhang et al., 2021).

**TABLE 3:** Fluorescence-based binding curves of resveratrol binding to SIRT1 in complex with different substrates. Data was fit to ligand depletion binding equation using GraphPad Prism. All measurements were performed in triplicate and the average and SEM are reported. Only the binding affinity of resveratrol to the SIRT1•ADPr•Ac-p53W complex is statistically significant different (*p-*value < 0.05) from the binding affinity of resveratrol to apo SIRT1.

Protein complex	*K*_D_ (μM)
apo SIRT1	28 ± 3.5
SIRT1•NAD^+^	19 ± 1.0
SIRT1•ADPr	16 ± 2.3
SIRT1•ADPr•Ac-p53W	84 ± 4.3
SIRT1•ADPr•Ac-p53	16 ± 6.0
SIRT1•ADPr•Ac-H4	24 ± 1.2
SIRT1•ADPr•Ac-H3	20 ± 3.2
SIRT1•ADPr•Ac-CSNK	28 ± 2.0

Previous work has shown that the binding affinity between resveratrol and SIRT1 could be influenced by adding peptide substrates. Interestingly, upon comparison of the binding affinity between resveratrol and the different SIRT1•substrate complexes, we found that resveratrol had a much weaker binding affinity to the SIRT1•ADPr•Ac-p53W complex, whereas it bound to all the other SIRT1 complexes with relatively similar binding affinity (Figures 5B, C; Table 3).

CABS-dock predictions of the peptide substrates binding to SIRT1 (Blaszczyk et al., 2016) suggest that the Rossman fold domain and a section of the zinc binding domain within the catalytic core undergo unique conformational changes upon binding of the p53W peptide that are not observed when the other peptide substrates are bound. Specifically, three β-strands in the Rossman fold are shifted and shortened, while there are two extended β-strands in the zinc-binding domain where there is originally a flexible loop, a region close to the resveratrol binding sites (Figure 5D).

## 4 Discussion

In this study, we compared the functional and conformational effects of a regulator on SIRT1 under both activation and inhibition scenarios. The data suggests that resveratrol has different interactions with and effects on SIRT1 in these two opposite scenarios. Enzyme kinetics results conclusively showed that the change in *K*_M_ values is the main driving force for both activation and inhibition effects, indicating that resveratrol affects the substrate recognition of SIRT1 regardless of the regulatory direction. This observation agrees with previous studies that found resveratrol-dependent conformational change of SIRT1 altered the substrate-enzyme binding interaction in activation scenarios (Borra et al., 2005; Kaeberlein et al., 2005; Hou et al., 2016), and further reveals that resveratrol inhibition also likely occurs through conformational change. However, our additional structural and functional studies suggest that the type of conformational change induced by resveratrol might differ in the two scenarios.

**FIGURE 6:**
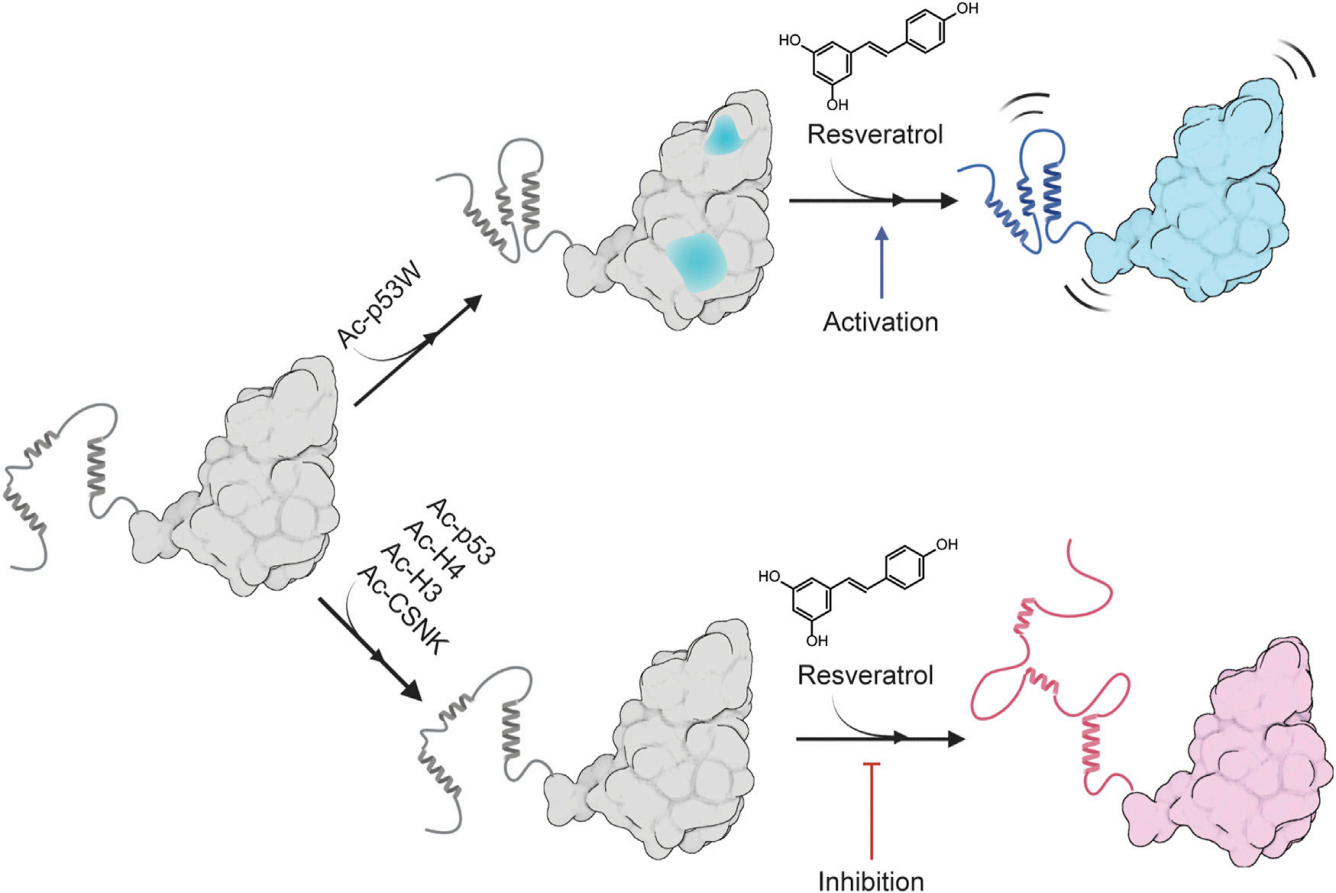
Proposed model for the peptide substrate-dependent regulation of resveratrol on SIRT1. A likely conformation of SIRT1 in solution is shown as gray, sections of SIRT1 that are predicted to undergo conformational change upon binding to Ac-p53W is shown in blue. The completely blue structure represents a more flexible SIRT1 that is activated by resveratrol, and the red structure represents a more disordered SIRT1 that is inhibited by resveratrol. Made using Biorender.com.

DSF experiments demonstrated that resveratrol destabilizes SIRT1 in both activation (towards Ac-p53W) and inhibition (towards Ac-p53, Ac-H4, Ac-H3 and Ac-CSNK) scenarios. Additionally, SAXS profiles showed that resveratrol extends the conformation of SIRT1 in both activation and inhibition scenarios. However, when resveratrol acts as an activator, the conformation of SIRT1 is evenly extended in both radius of gyration (R_g_) and radius of cross-section (R_xs_), whereas in the inhibition scenario, there is a larger change in R_xs_ compared to R_g_. We can propose that resveratrol renders SIRT1 more flexible in the activation scenario, allowing the enzyme to be more active and bind to a substrate more tightly by readily assuming a conformational change upon binding. On the other hand, in an inhibition scenario, resveratrol could significantly alter the protein conformation, resulting in weaker substrate binding. Indeed, previous protein NMR studies indicated that sections of the SBD become disordered upon binding of a protein inhibitor PACS-2 (Krzysiak et al., 2018). These possibilities are consistent with our observation that resveratrol destabilizes SIRT1 to a lesser extent in the activation scenario, as increased flexibility would cause minor destabilization compared to significant conformational change or unfolding. Similarly, increased protein flexibility in the resveratrol-as-activator scenario could reasonably lead to an extended protein conformation in both R_g_ and R_xs_, while order-to-disorder conformational changes in sections of the protein in the resveratrol-as-inhibitor scenario could potentially increase the cross-section more than just the overall volume (Figure 6).

What would cause resveratrol to have such different effects on SIRT1 based on the substrate sequence? With the knowledge that resveratrol might activate SIRT1 by stabilizing the protein-substrate interaction (Hou et al., 2016), it is tempting to assume that resveratrol would preferentially activate SIRT1 towards substrates with high *K*_M_ values. However, our data shows that SIRT1 has higher *K*_M_ values for all the peptide substrates in the resveratrol-as-inhibitor scenarios compared to the activator scenario. In regards to the peptide substrate sequences, it is worth noting that the +1 position is an aliphatic residue for all of the substrates in the resveratrol-as-inhibitor scenario, and is aromatic in the resveratrol-as-activator scenario. However previous studies have shown that resveratrol does not activate SIRT1 towards some acetyl-lysine substrates with aromatic residues in the +1 position either (Cao et al., 2015), suggesting that aliphatic versus aromatic properties at the +1 position might not be as significant a factor for resveratrol-dependent regulation.

The quantification of resveratrol binding affinity to SIRT1 in complex to different substrates was able to shed some light on this question. Intriguingly, the addition of peptide substrates from the resveratrol-as-inhibitor scenarios did not significantly affect resveratrol’s binding affinity to SIRT1, but the addition of Ac-p53W to SIRT1, where resveratrol acts as an activator, significantly compromised resveratrol’s binding affinity to the enzyme. Keeping in mind that previous crystal structures suggest that there are three possible binding sites in SIRT1 for resveratrol (Cao et al., 2015), our results could be explained by two possibilities: either 1) the binding of Ac-p53W to SIRT1 blocks the high-affinity resveratrol binding site or 2) alters the conformation of SIRT1, causing resveratrol to bind to a different, lower-affinity site. The first possibility is difficult to confirm without structural data, but CABS-dock models (Blaszczyk et al., 2016) for different peptide substrates interacting with SIRT1 indicate that p53W uniquely affects various structural elements of the protein, one of which are two extended β-sheets in an originally loop region close to the resveratrol binding sites. Whereas the other peptides have no such effect, supporting the second possibility. Additionally, we noticed that the Trp fluorescence emission spectrum of SIRT1 in complex with Ac-p53W and ADPr was noticeably different from the emission spectra of the other complexes: the emission peak was at 359 nm instead of the 330–337 nm range seen in other samples. While this could be attributed to the presence of a tryptophan residue within Ac-p53W, it is also possible that a unique change in SIRT1 structure contributes to this difference.

Overall, we propose a model for the differing regulatory effects of resveratrol on SIRT1 based on the substrate peptide, as illustrated in Figure 6. In brief, Ac-p53W in complex with SIRT1 could lead to changes in conformation or solvent accessibility, altering the way resveratrol binds to SIRT1. Conversely, the addition of the peptides in the resveratrol-as-inhibitor scenario has little to no effect on SIRT1 structure that could alter resveratrol binding. Since the interactions between resveratrol and SIRT1 in complex with various peptide substrates differ in nature, the resulting effect on SIRT1 is also different: in the activation scenario, resveratrol binding renders SIRT1 slightly more flexible, enhancing its efficiency, while in the inhibition scenario, resveratrol binding unravels the SBD helices of SIRT1, significantly compromising substrate recognition.

Of course, these are only preliminary proposed models, there is still a great deal of uncertainty surrounding the mechanism of SIRT1 regulation: it is not clear whether resveratrol or the substrate binds to the protein first or if the binding is sequential at all, and more detailed structural work must be carried out to test these proposed models. It is definitely a possibility that using the full-length substrate would give us more physiologically relevant results. However, the substrates in our study are either from the C-terminus (p53) or N-terminus (H3, H4, CSNK) of the protein and are relatively unstructured or in the case of p53, structured but is solvent exposed and has little contact with the rest of the protein. Hence we feel that the peptide substrates can give us a reasonable first approximation for the enzyme-substrate complex, given the difficulty of purifying multiple substrate proteins with single specific acetylate lysines. Additionally, this study only includes one peptide substrate in the resveratrol-as-activator scenario, as we were unable to experimentally confirm another peptide substrate where resveratrol acts as an activator. Several other peptide substrates where tested where resveratrol was identified as an activator in previous literature (Lakshminarasimhan et al., 2013). However in all those cases resveratrol either acted as an inhibitor or had no significant effect. This is possibly due to a difference in assay methods (literature used mass spectrometry based assay or microarray assay instead of the enzyme-coupled continuous assay in our study).

Nonetheless, these initial findings contribute to our understanding of the dual regulatory role of resveratrol on SIRT1 and could shed more light on the underlying mechanism for SIRT1’s allosteric regulation. This knowledge could also guide the development of targeted approaches to modulate SIRT1 activity for therapeutic purposes.

## Data Availability

The original contributions presented in the study are included in the article/Supplementary Material, further inquiries can be directed to the corresponding author.
