# Supplementary material for: The differing effects of a dual acting regulator on SIRT1
Source: Front Mol Biosci. 2023 Aug 30;10:1260489. doi: 10.3389/fmolb.2023.1260489 (PMC10499324; doi:10.3389/fmolb.2023.1260489)
Supplement: Supplementary file 1 [file DataSheet1.docx]

Supplementary Material

The Differing Effects of a Dual Acting Regulator on SIRT1

Yujin Hur^1^, Johnson Huynh^1^, Emily Leong^1^, Reena Dosanjh^1^, Annemarie Charvat^2^, My H. Vu^1^, Zain Alam^1^, Yuetong Lee^1^, Christiane C. Cabreros^1^, Emma C. Carroll^1^, Greg L. Hura^3^, Ningkun Wang^1*^

^1^Department of Chemistry, San José State University, San José, CA, USA

^2^Department of Pharmaceutical Chemistry, University of California San Francisco, San Francisco, CA, USA

^3^ Molecular Biophysics and Integrated Bioimaging Division, Lawrence Berkeley National Laboratory, Berkeley, CA, USA

*** Correspondence:**Ningkun Wang
ningkun.wang@sjsu.edu

# Supplementary Figures and Tables

## Supplementary Figures


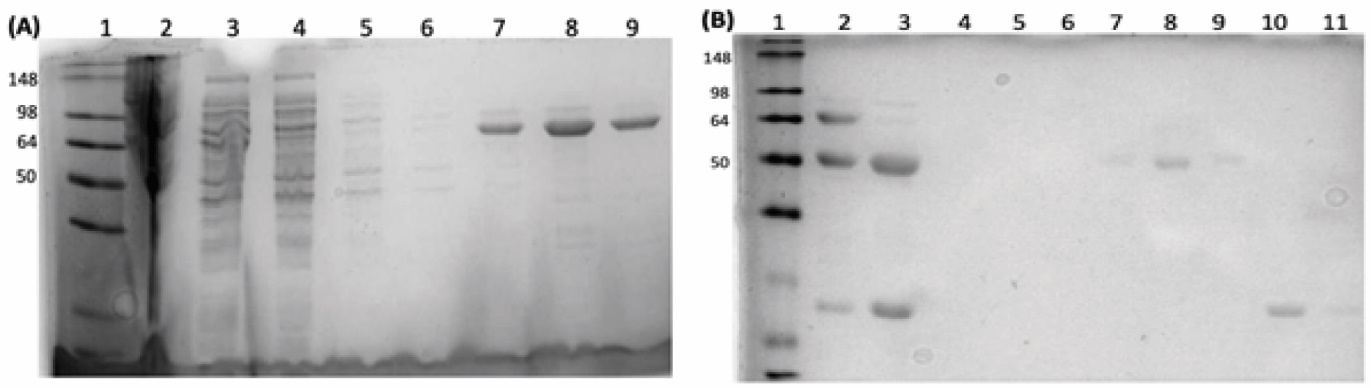


**Supplementary Figure 1.** (A) Coomassie Blue stained SDS-PAGE gel of hSIRT1-143 with SUMO tag: Lane 1: SeeBlue^TM^ Plus2 Pre-stained Protein Standard; Lane 2: Insoluble Lysate; Lane 3: Soluble Lysate; Lane 4: Flowthrough; Lane 5: Wash 1; Lane 6: Wash 2; Lane 7: Elution 1; Lane 8: Elution 2; Lane 9: Elution 3. (B) Coomassie Blue stained SDS-PAGE gel following purification using an SEC column and the cleavage of the SUMO solubility tag: Lane 1: SeeBlue^TM^ Plus2 Pre-stained Protein Standard; Lane 2: Pre-cleavage; Lane 3: Post-cleavage; Lane 4-11: FPLC fractions. hSIRT1-143 is at 46 kDa


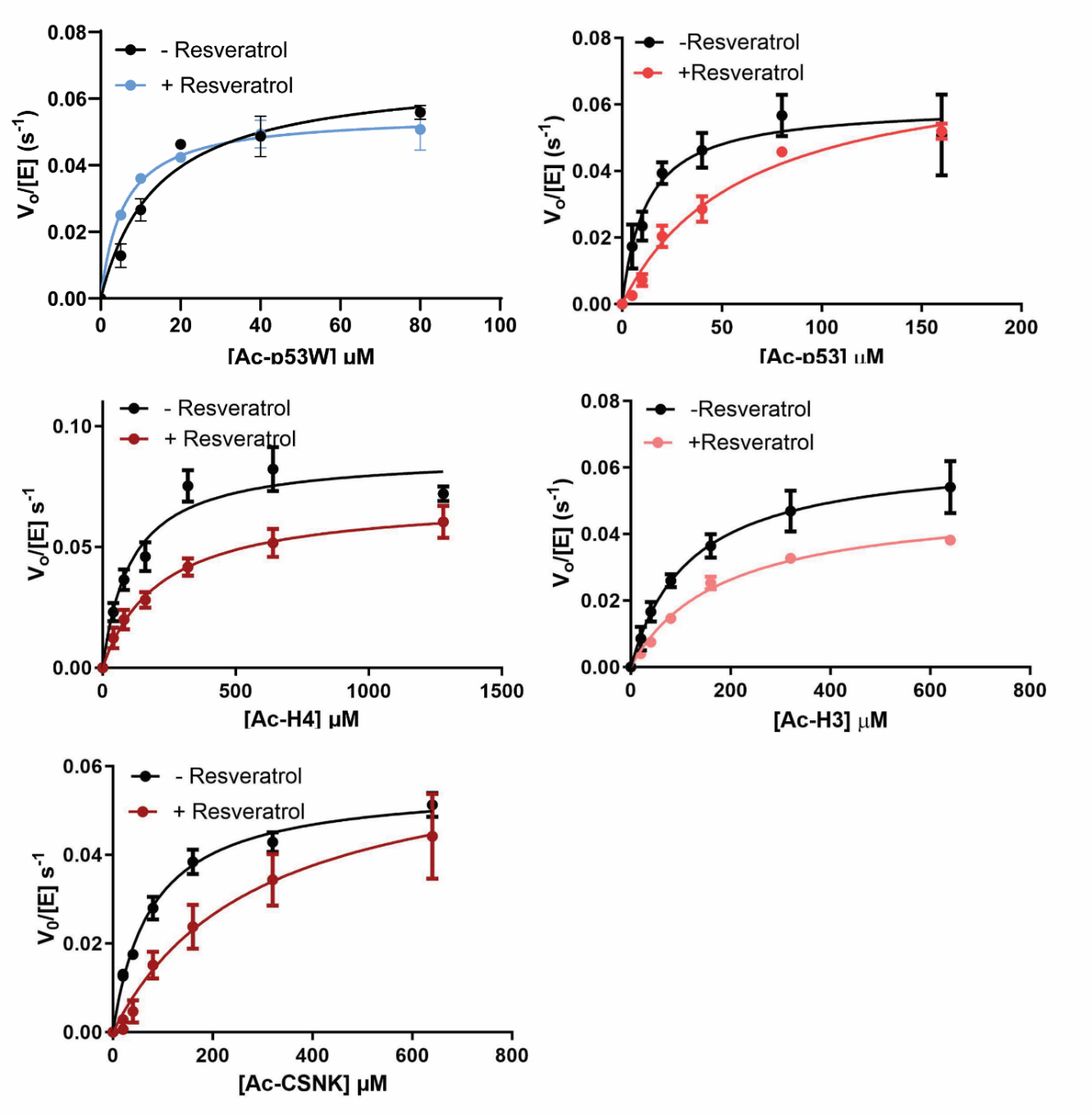


**Supplementary Figure 2.** Enzyme kinetics curves for SIRT1 activity against Ac-p53W, Ac-p53, Ac-H3, Ac-H4, and Ac-CSNK with and without the addition of resveratrol. Resveratrol acts as an activator for SIRT1 activity towards Ac-p53W and acts as an inhibitor for SIRT1 activity towards Ac-p53, Ac-H3, Ac-H4, and Ac-CSNK. All enzyme kinetics data were obtained in triplicates or duplicates. The Michaelis-Menten parameters were fit with GraphPad Prism.


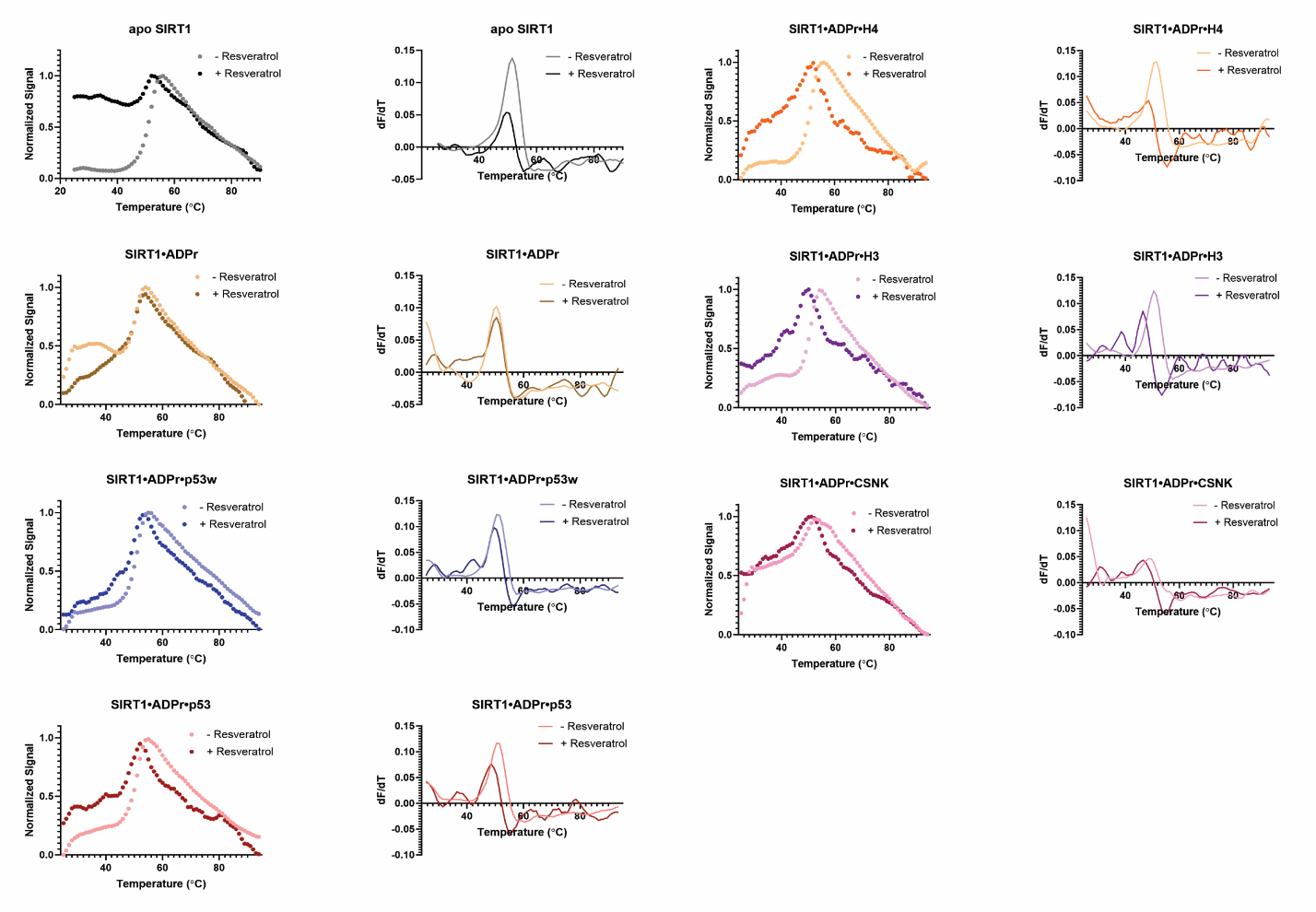


**Supplementary Figure 3.** Representative DSF melting curves and their first derivatives for apo SIRT1 and SIRT1•substrate complexes with and without the addition of resveratrol. Resveratrol acts as an activator for SIRT1 activity towards Ac-p53W and acts as an inhibitor for SIRT1 activity towards Ac-p53, Ac-H3, Ac-H4, and Ac-CSNK.


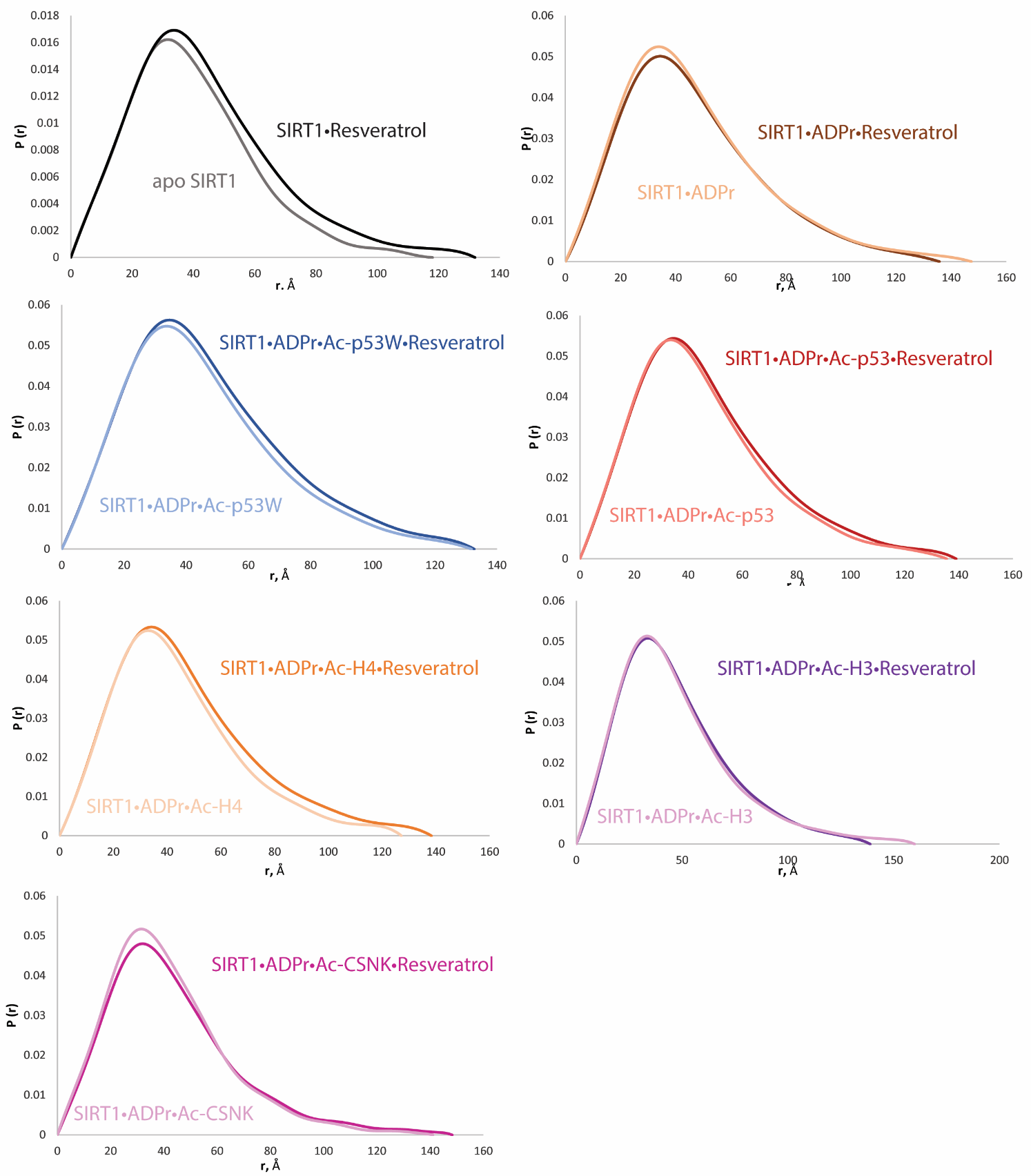


**Supplementary Figure 4.** Representative P(r) overlay graphs from ATSAS 3.0 Primus software for SIRT1•substrate complexes with and without the addition of resveratrol. The addition of resveratrol affects the extended population.


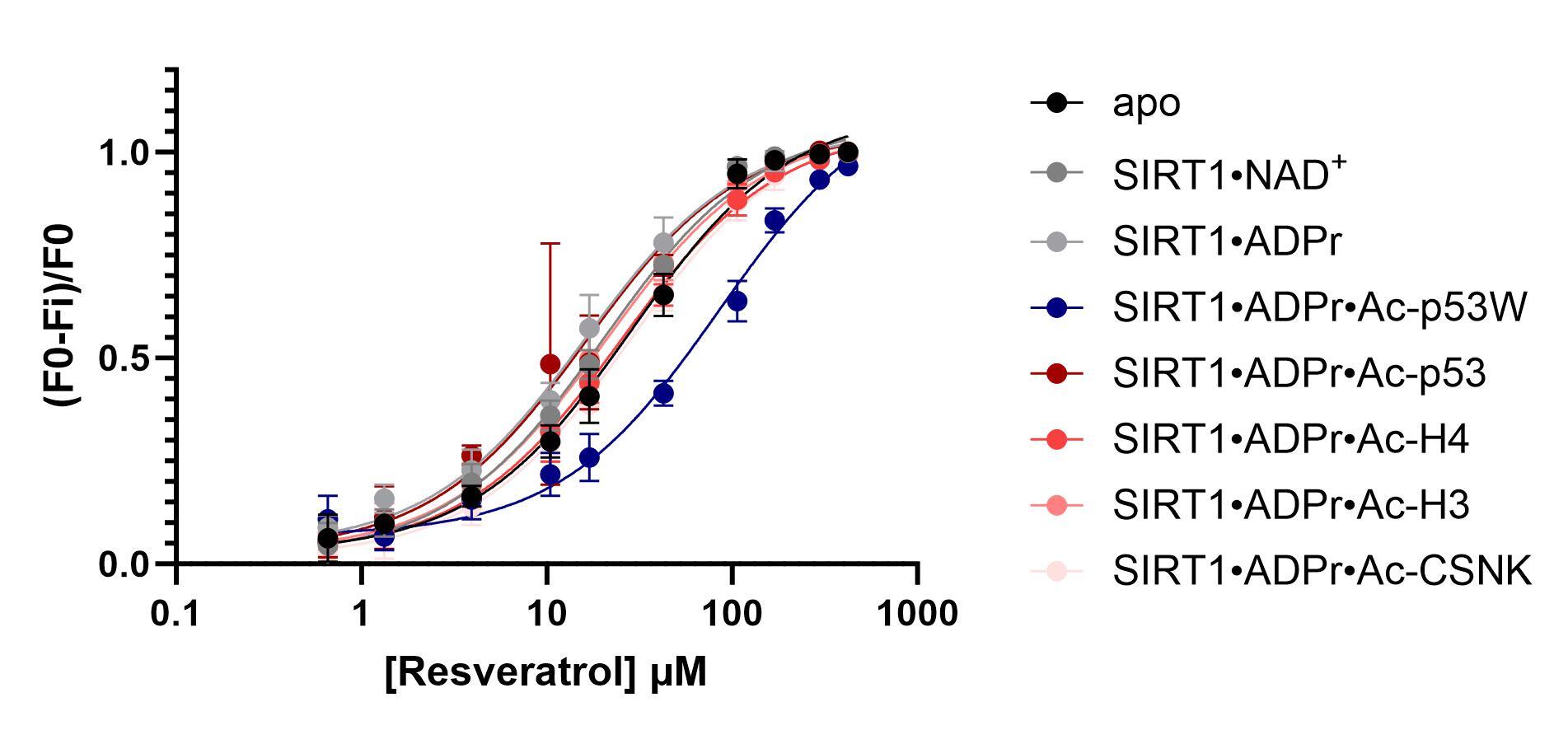


**Supplementary Figure 5.** Representative binding isotherms from GraphPad Prism for resveratrol binding to apo SIRT1 and SIRT1 in complex with substrates. Resveratrol acts as an activator for SIRT1 activity towards Ac-p53W and acts as an inhibitor for SIRT1 activity towards Ac-p53, Ac-H3, Ac-H4, and Ac-CSNK.

## Supplementary Table

**Supplementary Table 1.** The conformational behavior of SIRT1 in solution with peptide substrates and ADPr, with and without the addition of 200 μM resveratrol as determined by data collected at the Advanced Light Source SIBYLS beamline12.3.1 and analyzed using ATSAS 3.0 Primus software. All R_g_ and R_xs_ values are increased upon the addition of Resveratrol, R_xs_ values increase in a higher percentage than R_g_.

| Protein Complex | Resveratrol | R_g_ (Å) | Δ R_g_ (%) | R_xs_ | Δ R_xs_ (%) |
| --- | --- | --- | --- | --- | --- |
| apo SIRT1 | - | 30.35 | 6.3% | 10.26 | 9.7% |
|  | + | 32.26 |  | 11.26 |  |
| SIRT1•ADPr | - | 34.85 | 1.7% | 10.14 | 39.9% |
|  | + | 35.45 |  | 14.19 |  |
| SIRT1•ADPr•Ac-p53W | - | 33.9 | 8.9% | 12.97 | 6.9% |
|  | + | 36.91 |  | 13.86 |  |
| SIRT1•ADPr•Ac-p53 | - | 33.87 | 9.2% | 12.67 | 22.0% |
|  | + | 34.92 |  | 14.37 |  |
| SIRT1•ADPr•Ac-H4 | - | 33.88 | 7.3% | 11.23 | 18.7% |
|  | + | 36.99 |  | 13.7 |  |
| SIRT1•ADPr•Ac-H3 | - | 34.26 | 3.1% | 11.06 | 13.4% |
|  | + | 35.94 |  | 12.42 |  |
| SIRT1•ADPr•Ac-CSNK | - | 31.58 | 4.9% | 10.67 | 12.3% |
|  | + | 33.87 |  | 12.67 |  |
